# The value of serum Sema4D level in predicting the prognosis of patients with acute ST-segment elevation myocardial infarction and with high thrombus burden

**DOI:** 10.1186/s12872-023-03244-5

**Published:** 2023-05-03

**Authors:** Jie Bai, Liang Chen, Louyuan Xu, Qingquan Zhang, Jun Liu, Koulong Zheng

**Affiliations:** Department of Cardiology, Affiliated Hospital 2 of Nantong University, Nantong First People’s Hospital , Nantong, 226001 Jiangsu China

**Keywords:** sSema4D, Acute myocardial infarction, High thrombus burden, hs-CRP, MACE

## Abstract

**Background:**

Acute ST-segment elevation myocardial infarction (STEMI) is a serious cardiovascular disease. High thrombus burden is an independent risk factor for poor prognosis of acute myocardial infarction. However, there is no study on the correlation between soluble semaphorin 4D (sSema4D) level and high thrombus burden in patients with STEMI.

**Purpose:**

This study aimed to investigate the relationship between sSema4D level and the thrombus burden of STEMI and further explore its effect on the main predictive value of the occurrence of major adverse cardiovascular events (MACE).

**Methods:**

From October 2020 to June 2021, 100 patients with STEMI diagnosed in our hospital’s cardiology department were selected. According to the thrombolysis in myocardial infarction(TIMI)score, STEMI patients were divided into high thrombus burden groups (55 cases) and non-high thrombus burden groups (45 cases) 0.74 patients with stable coronary heart disease (CHD) were selected as stable CHD group, and 75 patients with negative coronary angiography (CAG) were selected as control group. Serum sSema4D levels were measured in 4 groups. The correlation between serum sSema4D and high-sensitivity C-reactive protein (hs-CRP) in patients with STEMI was analyzed. The relationship of serum sSema4D levels between the high and non-high thrombus burden group was evaluated. The effect of sSema4D levels on the occurrence of MACE was explored in one year after percutaneous coronary intervention.

**Results:**

Serum sSema4D level was positively correlated with hs-CRP level in STEMI patients (*P* < 0.05) with a correlation coefficient of 0.493. The sSema4D level was significantly higher in the high versus non-high thrombus burden group (22.54(20.82,24.17), *P* < 0.05). Moreover, MACE occurred in 19 cases in high thrombus burden group and 3 cases in non-high thrombus burden group. The results of Cox regression analysis showed that sSema4D was an independent predictor of MACE (OR = 1.497,95% CI: 1.213–1.847, *P* < 0.001).

**Conclusion:**

The sSema4D level is associated with coronary thrombus burden and is an independent risk factor for MACE.

## Introduction

Acute myocardial infarction (AMI) is the most common acute and severe cardiovascular disease, and has become a main cause of sudden death in adults owing to its acute onset, rapid progression, and high mortality rate [1]. The American Cardiovascular Research Center issued the “A Report of the American College of Cardiology/American Heart Association Joint Committee on Clinical Practice Guidelines ” [2] which stated that: the incidence of AMI is increasing annually and has become a major public health problem. The timely and effective opening of culprit blood vessels by percutaneous coronary intervention (PCI) or drug thrombolysis to improve myocardial blood supply is a common treatment for STEMI [3]. Numerous studies have confirmed that a high thrombus burden of infarct-related vessels is a high risk factor for poor outcomes following PCI. Accordingly, interventional therapy is bound to increase the risk of slow blood flow or no-reflow rate and even acute stent thrombosis, leading to a worsening patient prognosis and thereby increasing the risk of MACE [4, 5].

Sema4D/CD100 is a homodimeric protein that belongs to the semaphore family of axon-directing proteins. Members of the semaphore protein family recently received increasing attention because of their diverse functions in the immune system. Sema4D is the first semaphore with immune functions in T cell priming, antibody production, and intercellular adhesion play an important role [6]. Sema4D is expressed by most hematopoietic cells, including B and T lymphocytes, neutrophils, platelets, monocytes, and endothelial cells, and its expression typically increases upon cell activation. Among them, its expression level is highest in T cells, followed by neutrophils, platelets, and monocytes, all of which are involved in the pathogenesis of atherosclerosis [7, 8]. Under the stimulation of various inflammatory factors, Sema4D on the cell surface can be activated to form sSema4D [9, 10]. In atherosclerosis, sSema4D promotes platelet–platelet interactions and platelet and monocyte adhesion to endothelial cells, a key step in thrombus formation [11].

The relationship between sSema4D levels and chronic heart failure and coronary heart disease had been reported worldwide [12], but no reports have detailed sSema4D expression in STEMI patients or its relationship with coronary thrombus burden. In previous studies, it was demonstrated that cardiac troponin I (cTnI), high-sensitivity C-reactive protein (hs-CRP), and N-terminal precursor B-type brain natriuretic peptide (NT-proBNP) were associated with STEMI severity, but knowledge of those impacted on patient prognosis was limited [13].Therefore, it is of great clinical significance to actively explore the relevant indicators of thrombus burden and prognosis of patients with STEMI [14].

## Patients and methods

Participants A total of 100 patients with STEMI admitted to our hospital’s cardiology department between October 2020 and June 2021 were included. According to the TIMI score [15], STEMI patients were divided into a high thrombus burden group (55 patients) and a non-high thrombus burden group (45 patients). 74 patients with stable coronary heart disease(CHD)were selected as the stable CHD group, and 75 patients with negative coronary angiography (CAG) were selected as the control group.This study was approved by the local ethics committee (2019KN09) and all patients provided written informed consent.

The inclusion criteria were as follows: 18–75 years of age; having met the diagnostic criteria of the “2019 Chinese Society of Cardiology guidelines for the diagnosis and management of patients with ST-segment elevation myocardial infarction” [16]; The exclusion criteria were as follows: severe valvular heart disease; severe congenital heart disease; pulmonary heart disease; hypertrophic obstructive cardiomyopathy; hepatic insufficiency (defined as alanine transaminase or total bilirubin level greater than 3× the upper limit of normal); renal insufficiency (defined as a serum creatinine level greater than 1.5× the upper limit of normal); high bleeding risk; active peptic or skin ulcers; allergy to antiplatelet drugs; cardiogenic shock; malignant tumors; and coronary angiography showing left main disease.

### TIMI score

0 level, no thrombus ; 1 level, the lumen development was blurred ; 2 level, the length of thrombus was 1 / 2 of the diameter of the blood vessel ; 3level, the length of thrombus was 1 / 2–2 times the diameter of blood vessel ; 4 level, thrombus length > 2 times vessel diameter ; 5 level, completely occluded. High thrombus load is usually defined as greater than 2 level.

### Stable CHD

Stable CHD usually includes three conditions, namely chronic exertional angina, ischemic cardiomyopathy, and stable course phase after acute coronary syndrome (ACS) [17, 18].

### Negative coronary angiography

There was no obvious stenosis in the main coronary artery or its main branches.

### Treatment methods

All patients were administered aspirin 300 mg and clopidogrel 300 mg preoperatively. Postoperatively, the patients were administered nitrates, aspirin, clopidogrel, atorvastatin, β-blockers, and angiotensin-converting enzyme inhibitors according to the modern treatment of STEMI [19]。.

### Research methods

General patient information was also obtained. Immediately after admission, 2 mL of cubital venous blood was drawn from the patient, placed in a dry lithium heparin blood collection tube, and sent to our hospital’s biochemical laboratory for testing. Before surgery, 3 mL of blood was drawn from all patients and placed in dry lithium heparin blood collection tubes, centrifuged at 3000 × g for 15 min, and the supernatant was carefully collected in EP tubes with a pipette. The EP tubes were numbered, grouped together, and stored at -80 °C for testing. After the serum samples were collected uniformly, serum sSema4D levels were detected by a double-antibody one-step sandwich enzyme-linked immunosorbent assay (EK-H11846; Shanghai Enzyme Research Biotechnology Co., Ltd.) performed in strict accordance with the manufacturer’s instructions. Other routine laboratory tests were sent to our hospital’s biochemical laboratory (XN-9100 Automatic Blood Analyzer, Sysmex; LAboSPECT008AS automatic biochemical analyzer, Nanjing Sanhe Instrument Co., Ltd.; Multiskan FC microplate reader, Thermo; electrocardiograph, Naron Technology; and EPIQ5 color Doppler ultrasound system, Philips).

### sSema4D gene differential expression analysis in peripheral blood

Two datasets (GSE34198 and GSE48060) from the Gene Expression Omnibus (GEO) database were retrieved. The raw data were downloaded as MINiML files. The microarray data were normalized by the normalize quantiles function of the preprocessCore package in R software. Box plots were drawn using the “sva”R package, and the “ComBat” package was used to draw the principal component analysis (PCA) plot. The “sva” package [20] in R is often used to identify, estimate and remove the variation produced in high throughput gene expression microarray experiments to eliminate batch effects. After batch correction was completed, PCA was performed to assess whether the batch effects were eliminated. We extracted the sSema4D gene data from the two datasets and compared the difference in sSema4D gene expression in peripheral whole blood between the AMI and healthy control groups through the Wilcox test.

### Follow-up visit

All patients underwent electrocardiography immediately after admission and at 2 h postoperative. Each echocardiogram was reviewed in the outpatient clinic at 1 month postoperative and each patient followed up for more than 1 year after treatment. The occurrence of MACE and the time were recorded. MACE was considered as non-fatal ischemic or hemorrhagic stroke, non-fatal myocardial infarction, hospitalized unstable angina pectoris, unplanned revascularization including PCI and coronary artery bypass grafting (CABG), and cardiac death. Patients who died.

of non-cardiovascular diseases were divided into loss to follow-up.

The data were analyzed by SPSS version 23.0 statistical software. Quantitative normally distributed data are expressed as mean ± standard deviation ($\stackrel{-}{\text{X}}\pm$S). Non-normally distributed variables are represented as quartiles (P25, P75). Qualitative variables are expressed as frequencies. A t-test was used to compare the normally distributed enumeration data between groups, the chi-squared test was used to compare the categorical data between the groups, and a one-way analysis of variance was used to compare differences among multiple groups. Data that did not conform to a normal distribution were tested using a non-parametric test, while a binary logistic regression analysis was used to analyze the independent influencing factors of the binary factors. The receiver operating characteristic curve and area under the curve (AUC) were used to compare the diagnostic efficiencies of the different parameters, and the diagnostic sensitivity, specificity, and accuracy of each parameter were calculated. The Youden index was used to determine the best cutoff value. A multivariate Cox regression analysis was used to evaluate the effect of each parameter on survival, and the risk ratios and 95% confidence intervals were calculated. Spearman’s correlation was used for the correlation analysis, and values of *P* < 0.05 was considered statistically significant [11].

## Results

**Fig. 1:**
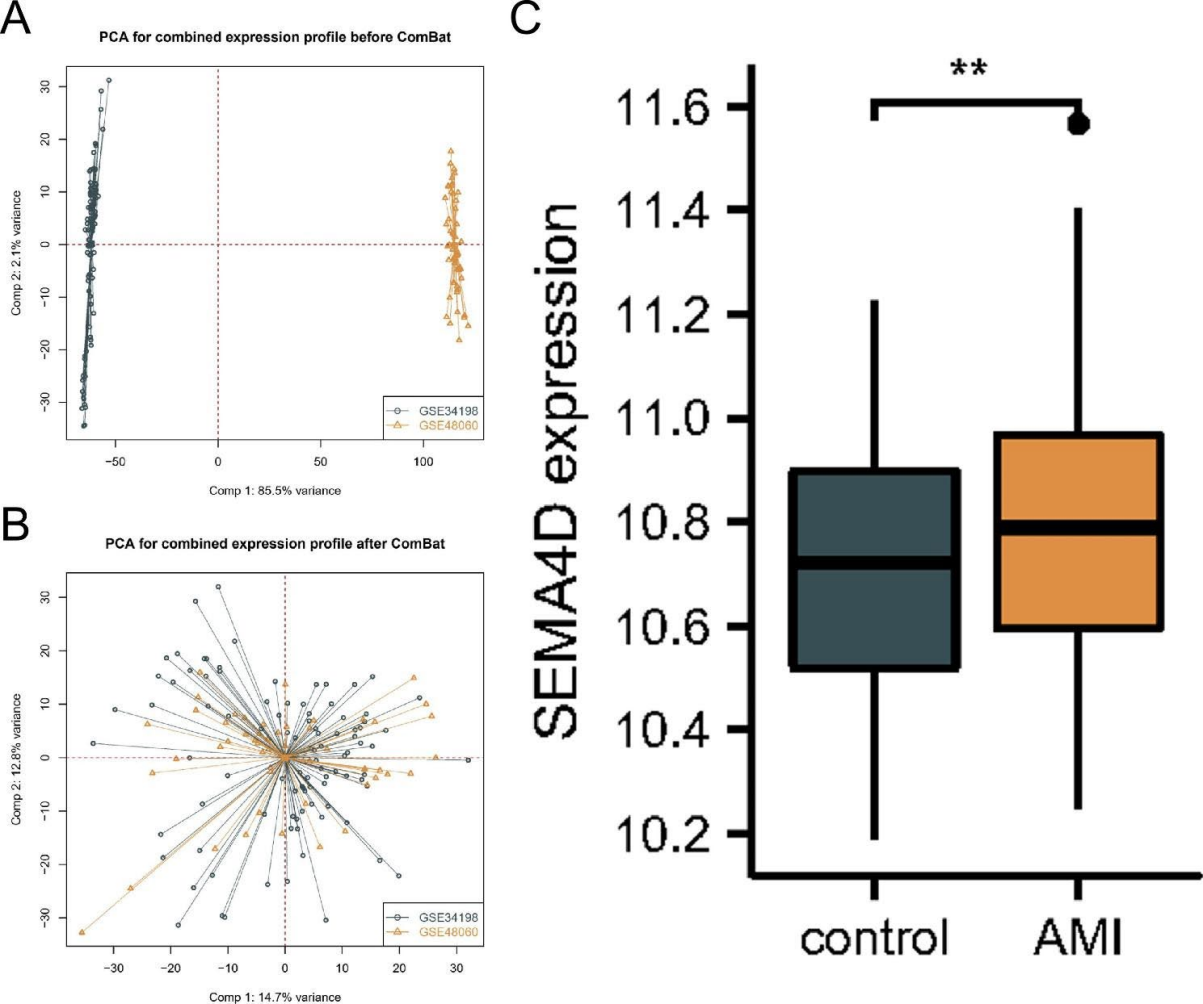
Principal component analysis (PCA) of gene expression datasets. (**A**) Gene expression profiles without the removal of the batch effect. (**B**) Gene expression profiles with removal of batch effect. (**C**) The expression levels of Sema4D between control and AMI.

The level of sSema4D in patients with STEMI was significantly higher than that in the control group (Fig. 1).

### Levels of sSema4D in each group

**Fig. 2:**
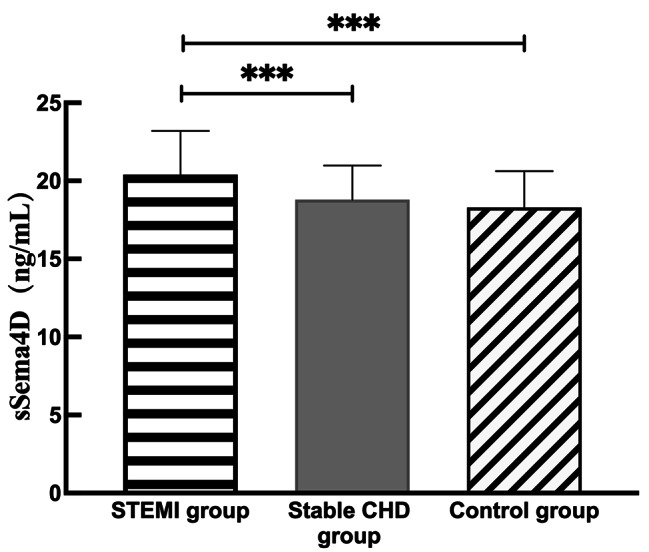
Comparison of serum sSema4D levels in the STEMI group, stable CHD group and control group. ***P < 0.001

Compared with the stable CHD group and the control group, sSema4D in the peripheral blood of the STEMI group was increased, and the difference was statistically significant (20.29(18.07,22.59), *P* < 0.001). There was no significant difference in serum sSema4D levels between the stable CHD group (18.38 (17.21,20.51) , *P* > 0.05) and the control group (17.93 (16.83,19.22) , *P* > 0.05) (Fig. 2).

### Correlation between serum sSema4D and hs-CRP levels in patients with STEMI

**Fig. 3:**
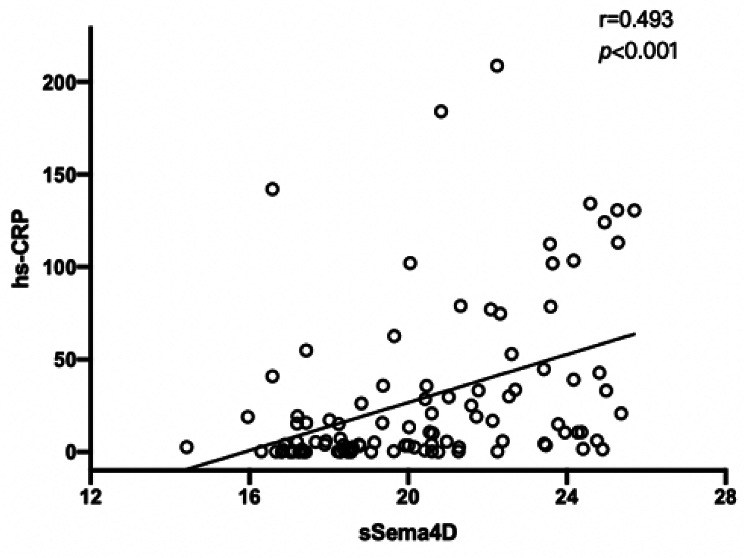
Correlation scatter plot of soluble semaphorin 4D (sSema4D) and high-sensitivity C-reactive protein (hs-CRP) levels in STEMI patients

Spearman’s correlation analysis was used to compare the correlation between the sSema4D and hs-CPR levels in patients with STEMI. The analysis revealed that sSema4D was positively correlated with hs-CRP (*P* < 0.001), with a correlation coefficient of 0.493 (Fig. 3).

### Correlation analysis of sSema4D and thrombus burden in STEMI

**Fig. 4:**
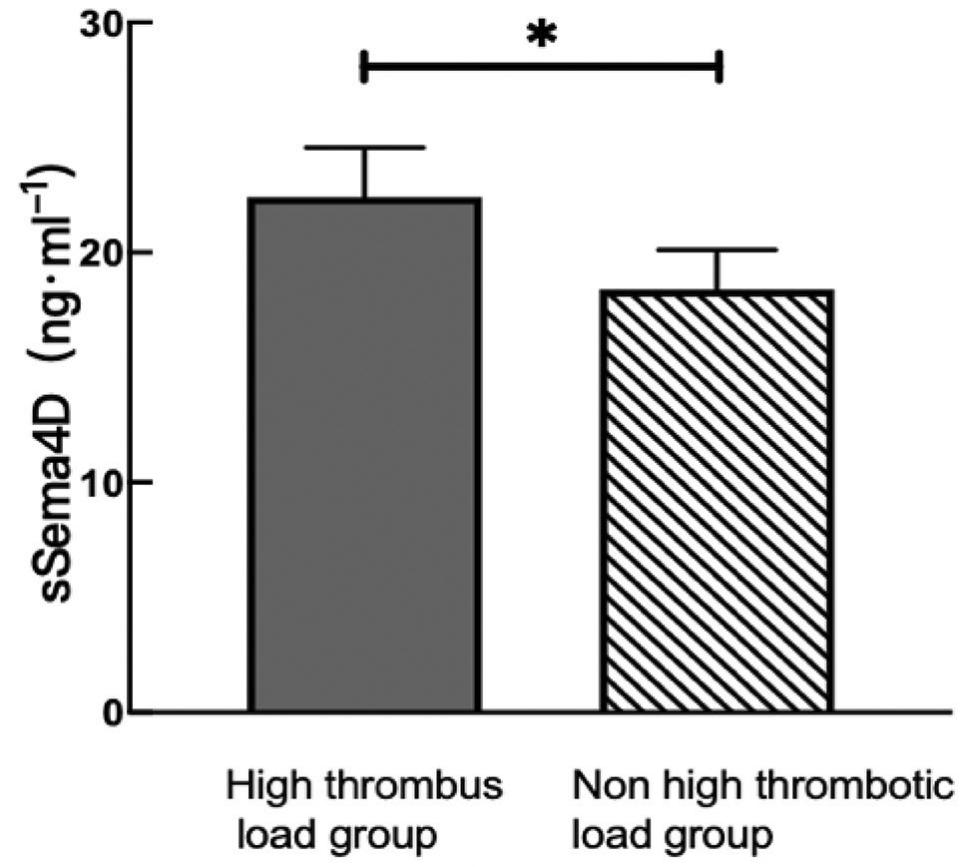
Serum sSema4D levels of high versus non-high thrombus burden groups*P < 0.05

The univariate analysis revealed no statistically significant intergroup differences in age, sex, hypertension, diabetes, smoking history, liver function (alanine transaminase level), renal function (creatinine level), low-density lipoprotein cholesterol, cardiac troponin I (cTNI), creatine kinase-MB (CK-MB), or other clinical indicators. The levels of total cholesterol, N-terminal precursor B-type brain natriuretic peptide (NT-proBNP)(1998.0(969.9,4407.5), *P* < 0.05), hs-CRP (25.16(20.00,67.00), *P* < 0.001) and sSema4D (22.54(20.82,24.17), *P* < 0.001) were higher in the high versus non-high thrombus burden group, while the left ventricular diastolic dysfunction (LVDd) (52.0(49.0,57.5), *P* < 0.05) and ST-segment.

regression rate(22.54(20.82,24.17), *P* < 0.001) were lower in the high versus non-high thrombus burden group (Table 1; Fig. 4).

**Table 1:** Baseline characteristics and sSema4D levels of the high versus non-high thrombus burden groups

Variable	high thrombus burden	non-high thrombus burden	*P* value
Age (y)	67.0(54.0,73.5)	61.0(52.0,71.0)	0.188
Gender, n (Male %)	24(42.9)	32(57.1)	0.627
Hypertension, n (%)	34(59.6)	23(40.4)	0.282
Diabetes, n (%)	16(55.2)	13(44.8)	0.191
Smoking, n (%)	26(49.1)	27(50.9)	0.387
ALT (U/L)	37.0(20.0,67.0)	27.0(18.0,45.0)	0.060
Cr (umol/L)	65.81 ± 19.55	70.14 ± 17.77	0.249
TC (mmol/L)	4.81 ± 1.29	4.11 ± 1.30	0.008
LDL-C (mmol/L)	3.02 ± 1.25	2.85 ± 0.87	0.447
NT-proBNP (pg/ml)	1998.0(969.9,4407.5)	1409.0(542.9,2428.0)	0.019
hs-CRP (mg/L)	25.16(20.00,67.00)	4.01(0.44,15.69)	< 0.001
CK-MB (ng/ml)	81.80(24.15,281.40)	97.00(25.30,246.20)	0.843
cTNI (ng/ml)	38.90(7.98,80.00)	21.00(3.42,67.40)	0.203
LVDd (mm)	52.0(49.0,57.5)	51.0(47.0,53.0)	0.046
ST-segment regression(%)	50.0(25.0,67.0)	75.0(63.0, 100.0)	< 0.001
sSema4D (ng/ml)	22.54(20.82,24.17)	18.37(17.21,19.65)	< 0.001

ALT, alanine aminotransferase; CK-MB, creatine kinase-MB; Cr, creatinine; cTNI, cardiac troponin I; hs-CRP, high-sensitivity C-reactive protein; LDL-C, low-density lipoprotein cholesterol; LVDd, left ventricular diastolic dysfunction; NT-proBNP, N-terminal precursor B-type brain natriuretic peptide; sSema4D, soluble semaphorin 4D; TC, total cholesterol.

### Binary logistic regression analysis for evaluating independent predictors of STEMI

A multivariate binary logistic regression analysis showed that sSema4D was an independent risk factor for a high thrombus burden in STEMI (odds ratio(OR), 1.938; 95% confidence interval(CI), 1.442–2.604; *P* < 0.001; Table 2).

**Table 2:** Binary logistic regression analysis of factors related to thrombus burden in STEMI

Factor	OR	95%CI		P value
TC	1.336	0.852	2.093	0.207
NTpro-BNP	1.000	1.000	1.000	0.720
hs-CRP	1.002	0.987	1.017	0.816
LVDd	1.022	0.933	1.118	0.643
ST-segment regression rate	7.042	0.617	83.333	0.116
sSema4D	1.938	1.442	2.604	< 0.001

CI, confidence interval; hs-CRP, high-sensitivity C-reactive protein; LVDd, left ventricular diastolic dysfunction; NT-proBNP, N-terminal precursor B-type brain natriuretic peptide; OR, odds ratio; TC, total cholesterol.

### Receiver operating characteristic curve for evaluating predictive value of sSema4D in STEMI with high thrombus burden

**Fig. 5:**
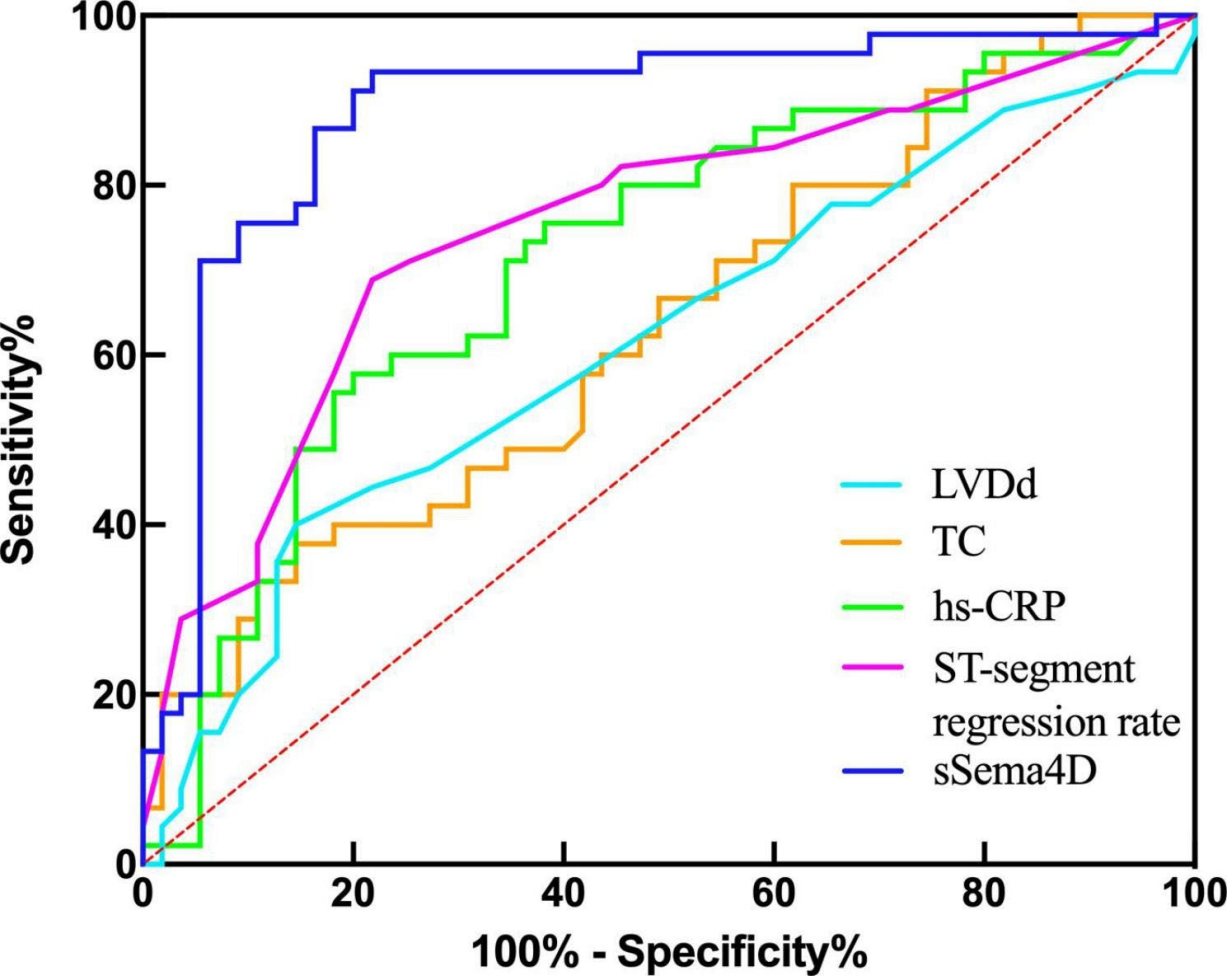
Receiver operating characteristic curve analysis of left ventricular diastolic dysfunction (LVDd), total cholesterol (TC), high-sensitivity C-reactive protein (hs-CRP), ST-segment regression rate, and soluble semaphorin 4D (sSema4D)

Receiver operating characteristic curves were drawn (Fig. 5; Table 3) to compare the predictive value of TC, hs-CRP, LVDd, ST-segment regression rate, and sSema4D level for a high thrombus burden in STEMI. Among them, hs-CRP, ST-segment regression rate, and sSema4D had predictive value (*P* < 0.05); the AUC of sSema4D was 0.888, while the cutoff value was 19.968. At this time, its specificity of predicting STEMI thrombus burden was 78.2%, while the sensitivity was 93.3%.

**Table 3:** Comparison of clinical characteristics and predictive value of sSema4D in STEMI patients

Factor	AUC	95%CI		*P* value	CUT-OFF	Sensitivity	Specificity
TC	0.633	0.524	0.742	0.023	5.385	37.8%	85.5%
hs-CRP	0.715	0.612	0.817	< 0.001	19.050	57.8%	80.0%
LVDd	0.616	0.503	0.729	0.047	54.5	40%	85.5%
ST-segment repression rate	0.753	0.655	0.852	< 0.001	0.615	68.9%	78.2%
sSema4D	0.888	0.816	0.959	< 0.001	19.968	93.3%	78.2%

AUC, area under the curve; CI, confidence interval; hs-CRP, high-sensitivity C-reactive protein; LVDd, left ventricular diastolic dysfunction; sSema4D, soluble semaphorin 4D; TC, total cholesterol.

### Independent predictors of MACE

Univariate Cox regression analysis revealed that NT-proBNP, hs-CRP, ejection fraction (EF), LVDd, ST-segment regression rate, and sSema4D level were prognostic factors in STEMI patients (all *P* < 0.05). In the multivariate Cox regression analysis, we found that sSema4D and ejection fraction were independent factors affecting the prognosis of STEMI (HR = 1.497, 95%CI: 1.213–1.847, *P* < 0.001) (Table 4).

**Table 4:** Univariate and multivariate Cox regression analysis of prognostic factors for ST-segment elevation myocardial infarction

Variables	Univariate analysis				Mulitivariate anaiysis		
	Hazard Ratio	95%CI	*P* value		Hazard Ratio	95%CI	*P* value
Age	1.008	0.974–1.043	0.654				
Gender	0.858	0.367–2.007	0.724				
Hypertension	0.759	0.318–1.810	0.534				
Smoking	1.138	0.493–1.810	0.762				
Diabetes	1.154	0.493–2.951	0.765				
NT-proNBNP	1.000	1.000–1.000	0.001				0.345
ALT	1.001	0.995–1.007	0.673				
Cr	1.014	0.991–1.036	0.232				
hs-CRP	1.010	1.003–1.017	0.004				0.546
LDL-C	1.027	0.693–1.522	0.895				
TC	1.268	0.935–1.720	0.126				
EF	0.885	0.840–0.933	0.000		0.915	0.867–0.966	< 0.05
LVDd	1.069	1.014–1.127	0.013				0.377
ST-segment regression rate	0.061	0.013–0.302	0.001				0.504
sSema4D	1.652	1.347–2.025	0.000		1.497	1.213–1.847	< 0.001

ALT, alanine transaminase; CI, confidence interval; Cr, creatinine; hs-CRP, high-sensitivity C-reactive protein; EF, ejection fraction; LDL-C, low-density lipoprotein cholesterol; LVDd, left ventricular diastolic dysfunction; NT-proBNP, N-terminal precursor B-type brain natriuretic peptide; sSema4D, soluble semaphorin 4D; TC, total cholesterol.

## Discussion

This exploratory study evaluated the predictive value of sSema4D level and different thrombus burdens in patients with STEMI and its correlation with prognosis. This study evaluated the relationship between sSema4D and prognosis from various aspects and time periods and avoided the lack of rigor of a single factor.

STEMI is a common cardiac emergency that manifests as ischemic chest pain, fever accompanied by tachycardia, frequent angina pectoris, and heart failure in severe cases [21]. An estimated 30–37% of cases involve postoperative slow reflow or no-reflow rate with a 9–15% distal embolization rate after PCI treatment, indicating limited clinical benefit of PCI [22]. The slow blood flow and no-reflow of myocardial tissue caused by a high thrombus load within the coronary artery has become the largest obstacle to effective myocardial reperfusion in the treatment of STEMI, which seriously affects patient prognosis [23]. The correct and effective assessment and management of lesions with high thrombus burden are key issues to further improving the short- and long-term prognosis of STEMI patients. Tirofiban and thrombus aspiration are currently used to treat STEMI with a high thrombus burden to a certain extent, but such treatment is ineffective in some cases, making it difficult to meet clinical needs [24, 25].

sSema4D was originally discovered in the immune system and it regulates B cell aggregation, survival and T cell activation [26].sSema4D binds to multiple receptors, plexin-B1/B2, CD72, and plexin C1, which mediate its effects on neural, immune, endothelial, and epithelial cells; therefore, with immune system activation, angiogenesis, bone metabolism, and neural development play a pleiotropic effect [27–31]. Sema4D is expressed in human atherosclerosis, more specifically by plaque macrophages and foam cells [11]. Mou [32] reported a dual mechanism in the absence of Sema4D-mediated platelet activation and aggregation. Since platelets can express both Sema4D and PlexinB1, Sema4D that initially attaches to the platelet surface can act as a ligand for platelet–platelet interactions to initiate coupling and then activate tyrosine kinase to induce platelet activation, thereby promoting thrombosis [33]. Sema4D knockout can inhibit platelet aggregation and thrombus growth and improve prognosis. Previous studies reported that Sema4D can mediate the PlexinB1 pathway to indirectly promote thrombosis [34]. Further examination of the Sema4D/PlexinB1 pathway may provide new ideas for the diagnosis and treatment of cardiovascular events caused by atherosclerosis and improve patient prognosis.

Activation of the inflammatory response is an important pathological change in cardiovascular and cerebrovascular diseases, while atherosclerotic plaque formation and rupture are closely related to myocardial cell and post-stent injuries [35]. It is particularly important to explore the correlation between sSema4D-related inflammatory factors, disease progression, and prognosis. The results of this study demonstrated a higher probability of a poor prognosis in patients in the high versus low sSema4D group and that the risk of MACE after PCI was also extremely high. Previous studies reported that sSema4D and hs-CRP levels may be independent risk factors for coronary heart disease [11]. Here we concluded that sSema4D levels are highly expressed in patients with a high thrombus burden and correlated with hs-CRP levels (r = 0.493, *P* < 0.001). We infer that sSema4D, like hs-CRP, mediates the inflammatory response and participates in the arteriosclerosis process of STEMI [36]. In many inflammatory autoimmune diseases, such as rheumatoid arthritis [37], anti-neutrophil cytoplasmic antibody-associated vasculitis [9] and coronary artery stenosis [11], elevated serum sSema4D levels have been reported. By binding to different receptors, sSema4D exhibits different effects on the inflammatory phenotypes of different types of cells. For example, the Sema4D-plexin B1 axis promotes chondrocyte inflammation [38] while the Sema4D-plexin B2 axis promotes keratinocyte inflammation [39]. Interleukin (IL)-1, IL-2, IL-6, and tumor necrosis factor α are several types of tissues and cells involved in the inflammatory process, including leukocytes, platelets, tissue macrophages, and endothelial cells [40, 41]. These chemokines affect T lymphocytes by regulating different functions of T lymphocytes. This special inflammatory pathway can explain the increased secretion of Sema4D in STEMI. Recent reports suggest that sSema4D is an inducer of some proinflammatory cytokines and is involved in endothelial inflammation and vascular dysfunction [9, 29].However, no specific research has proven the mechanism of sSema4D participation, and further studies are needed to explore its involvement in atherosclerosis formation by mediating inflammatory responses.

In the past, cTnI was known to have high sensitivity and specificity for the diagnosis and evaluation of slight myocardial injury. Nageh [42] and others reported that an increased cTnI level after PCI is an important indicator of prognosis, indicating the risk of MACE. However, cTnI levels may also be elevated in sepsis, myocarditis, or unrelated myocardial injury. When AMI occurs, to maintain its own function, blood pressure, and blood volume, the heart increases its secretion of B-type natriuretic peptide (BNP) by ventricular myocytes, increasing the plasma BNP level. The increase in BNP was positively correlated with degree of myocardial infarction. Numerous experiments have proven that the higher the BNP concentration, the greater the probability of MACE such as LVDd after PCI [43]. However, it remains uncertain at which stage the BNP measurement is more valuable for predicting MACE after PCI.

On the one hand, hs-CRP can activate the complement system, release harmful terminal products, and damage the myocardium. However, as an inflammatory factor, it has a chemotactic effect on fibrin, which can lead to thrombosis [44]. During stent implantation in patients undergoing PCI, stimulation of the vascular wall by the stent (as a foreign body) can damage the vascular endothelium and smooth muscle, thereby aggravating the inflammatory response and leading to in-stent restenosis [45]. Some studies have proven that hs-CRP has important predictive value for the occurrence of MACE [46], but because of its low sensitivity and specificity, clinical references are few.

In recent years, with increasing clinical research, reperfusion at the cellular level in patients after PCI can be identified with a change in the occlusion site; that is, the presence of a ST-segment change on the electrocardiogram can predict prognosis. In this experiment, the comparison of ST-segment regression rate values between the high and non-high thrombus burden groups of STEMI patients was investigated at 2 h postoperative. It was concluded that the fallback ratio of non-high thrombus burden group was larger (75.0(63.0, 100.0), *P* < 0.05), that is, the lower the expression level of sSema4D, the better the fallback situation and the better the prognosis of patients. However, Chinese studies on ST-segment regression after reperfusion therapy primarily used resting electrocardiograms, which are mostly intermittent recordings that cannot continuously observe ST-segment changes or provide long-term monitoring of arrhythmias; therefore, their usefulness is limited.

This study also found that sSema4D was an important risk factor for MACE after PCI that was closely related to prognosis (HR = 1.497, 95%CI: 1.213–1.847,*P* < 0.001). This is because sSema4D is involved in biological processes such as cell migration and angiogenesis. Its expression level decreases with STEMI coronary remodeling, participates in the pathogenesis of myocardial ischemia, and can have a synergistic effect with serum inflammatory factors, which together lead to disease deterioration. Moreover, it is directly involved in the cardiac signaling pathway or indirectly involved in the myocardial-independent pathway. In this study, the shortest time to MACE events was 1 year, which is relatively limited; moreover, its sample size is small and unable to fully reflect the long-term occurrence of MACE. During the follow-up process, patients reported having a vague memory of the specific timing of the occurrence of MACE events; sSema4D may be more effective than myocardial markers in the STEMI with high thrombus load, providing new ideas for the prevention and treatment of STEMI with high thrombus load and becoming a potential therapeutic target. Thus, the results may exhibit deviations. In future studies, we will further investigate the specific mechanism by which sSema4D affects coronary thrombus burden.

## Conclusions

This study revealed that sSema4D in STEMI patients was significantly expressed in the high thrombus burden group(22.54(20.82,24.17), *P* < 0.001); moreover, it was an independent predictor of MACE in the Cox regression analysis (HR = 1.497,95% CI: 1.213–1.847, *P* < 0.001).

. It can be inferred that sSema4D is a potential therapeutic target for patients with a high thrombus burden and may serve as a new inflammatory marker for coronary atherosclerosis.

## Data Availability

The datasets used and/or analyzed during the current study are available from the corresponding author upon reasonable request.
